# Agro-Industrial Fruit Byproducts as Health-Promoting Ingredients Used to Supplement Baked Food Products

**DOI:** 10.3390/foods11203181

**Published:** 2022-10-12

**Authors:** B. Shain Zuñiga-Martínez, J. Abraham Domínguez-Avila, R. Maribel Robles-Sánchez, Jesus Fernando Ayala-Zavala, Mónica A. Villegas-Ochoa, Gustavo A. González-Aguilar

**Affiliations:** 1 Centro de Investigación en Alimentación y Desarrollo A. C. Carretera Gustavo Enrique Astiazarán Rosas No. 46, Col. La Victoria, Hermosillo 83304, Sonora, Mexico; 2 CONACYT-Centro de Investigación en Alimentación y Desarrollo A. C. Carretera Gustavo Enrique Astiazarán Rosas No. 46, Col. La Victoria, Hermosillo 83304, Sonora, Mexico; 3 Departamento de Investigación y Posgrado en Alimentos, Universidad de Sonora, Blvd. Luis Encinas y Rosales s/n, Col Centro, Hermosillo 83000, Sonora, Mexico

**Keywords:** fruit processing, pomace, bioactive, compounds, agroindustrial waste, natural additives, glycemic index, sensory acceptance

## Abstract

One of the biggest problems faced by food industries is the generation of large amounts of agro-industrial byproducts, such as those derived from fruit processing, as well as the negative effects of their inadequate management. Approximately 1/3 of the food produced worldwide is unused or is otherwise wasted along the chain, which represents a burden on the environment and an inefficiency of the system. Thus, there is growing interest in reintroducing agro-industrial byproducts (both from fruits and other sources) into the processing chain, either by adding them as such or utilizing them as sources of health-promoting bioactive compounds. The present work discusses recent scientific studies on the nutritional and bioactive composition of some agro-industrial byproducts derived from fruit processing, their applications as ingredients to supplement baked foods, and their main biological activities on the consumer’s health. Research shows that agro-industrial fruit byproducts can be incorporated into various baked foods, increasing their fiber content, bioactive profile, and antioxidant capacity, in addition to other positive effects such as reducing their glycemic impact and inducing satiety, all while maintaining good sensory acceptance. Using agro-industrial fruit byproducts as food ingredients avoids discarding them; it can promote some bioactivities and maintain or even improve sensory acceptance. This contributes to incorporating edible material back into the processing chain as part of a circular bioeconomy, which can significantly benefit primary producers, processing industries (particularly smaller ones), and the final consumer.

## 1. Introduction

The food processing industry generates a large amount of waste and byproducts, which is continuously increasing due to industrialization and urbanization [1]. An estimated 221 million tons of food are lost and wasted yearly along the production and retail chains [2]. The byproducts of their transformation can represent 25 to 60% of the weight of a fruit, for example, fruits that are usually marketed in the form of paste, juice, or sauce, in which the seeds, peel, and their combined fraction (pomace) must be separated, resulting in a significant volume of byproducts [3]. It has become increasingly clear in recent years that the linear use of resources (production, consumption, and generation of waste) is not sustainable without negative consequences; therefore, a circular bioeconomy model has been considered as a way to use natural resources efficiently and to decrease the amount of unused or wasted biomass. The valorization of these byproducts and waste will reduce environmental pollution and lead to value-added products, thus creating potential subsidiary markets and sources of income [4].

Although agro-industrial byproducts are generally used as animal feed, compost, or simply discarded, they can have other important roles since, during the last decade, various works have shown that they are sources of bioactive compounds, such as phenolic compounds, antioxidants, dietary fiber, carotenoids, natural pigments, and protein, among others. This composition can be used to develop new and sustainable functional foods with potentially high nutritional value [3,5,6]. The search for natural bioactive compounds is also a priority for the management and prevention of diseases because they can promote health through several mechanisms of action, such as the ability to interact with proteins, DNA, and other biological molecules, as well as the potential to stop free-radical induced oxidation, potential to modify the composition and metabolic activity of the intestinal microbiota, and regulation of serum lipid and glucose levels, among others [7]. These components are an excellent set of molecules for the production of nutraceuticals, functional foods, and food additives, coupled with the growing interest from consumers in acquiring healthy products made from natural ingredients that can mitigate their risk of disease [8].

An interesting strategy to increase the intake of these compounds is to develop functional foods based on traditional high-consumption products, such as baked products. Depending on their ingredients, these foods can be energy-dense and high in simple carbohydrates and fats (including saturated and *trans*), whose regular consumption can lead to various non-communicable diseases. Thus, a wide variety of baked products like cookies/biscuits, corn chips, extrudates, breadsticks, cakes, and muffins, among others, have been developed using different types of byproducts to supplement them and counter some of the aforementioned negative properties, leading to improvements of their nutritional content. However, adding byproducts has been challenging for food scientists since their presence can lead to undesired changes in the final product’s physicochemical and sensory properties. This can be caused by the byproducts’ complex matrix that promotes different interactions and the presence of molecules with strong sensory profiles, which has been recently reviewed elsewhere [9,10]. Aspects such as byproduct origin, particle size, water retention capacity, and gel-forming ability, among others, are some of the byproducts’ variables that can be of relevance to the final product’s physicochemical profile; their impact will also depend on the specific product that is to be supplemented. Furthermore, they can impart flavors, odors, and strong colors, affecting the product’s sensory profile and consumer perception. This makes it necessary to carefully select the best percentages of addition [11] and choose the formulation that can potentially promote beneficial bioactivities while preserving or enhancing the sensory qualities of the original product [12]. Such analyses can sometimes suffice, but additional pretreatments may also be required, depending on the specific byproduct in question.

The present work aims to discuss recent scientific studies on the nutritional, sensory, and bioactive composition of some baked products supplemented with agro-industrial byproducts of the fruit industry due to their potential as economically viable sources of health-promoting bioactive compounds. Baked foods are of particular interest because they are commonly made with refined flours and other ultra-processed ingredients; thus, supplementing them can increase their nutritional value. These findings are intended to contribute to future research on new functional foods based on other agro-industrial byproducts, which could exert bioactivities that promote consumers’ health.

## 2. Agro-Industrial Fruit Byproducts: Origin and Potential Source of Bioactive Compounds

Before considering the potential applications of agro-industrial fruit byproducts, they must first be described. This section will briefly mention their origins and highlight some compounds that make them desirable to be reincorporated into the food chain. Agro-industrial byproducts (from fruits or other vegetable sources) are mainly composed of seeds, peels, stems, leaves, and unusable pulp, and are generated at all stages of the food supply chain, i.e., during agricultural production, processing, and distribution. Food waste is also generated in parallel, and a particular byproduct may also be considered wasted food, for example, fruit pulp that is unused in a final product but which is otherwise safe for consumption. Up to 42% occurs in domestic activities, 39% in the food manufacturing industry, 14% in the food service sector (ready-to-eat food and restaurants), and 5% during distribution [13]. It should be noted that the proportion of byproducts generated at each stage varies significantly from country to country, depending on the level of development of the regions where the crops are grown. For example, in developing countries, harvesting and processing are the most important stages contributing to the generation of byproducts, with a share of approximately 50% of initial food production. Premature harvesting, poor storage facilities, processing, and lack of infrastructure are among the main causes of this high proportion, while the consumption stage generates less than 10% of losses [13,14]. In contrast, the major food losses (around 40%) in industrialized countries are usually associated with the end of the supply chain (retail and consumption stages). The generation of byproducts in industrialized regions is also related to the highly demanding quality standards set by retailers and requested by consumers in terms of the shape, size, and color of products [15,16]. Therefore, the total amount of byproducts in industrialized countries is also significant, despite relatively low losses during post-harvest and processing of raw materials.

Byproducts can be a significant source of environmental pollution since they have a high biodegradability associated with their high moisture content that favors high microbial loads [17]. Anaerobic biological degradation of organic matter is the third largest anthropogenic source of atmospheric methane emissions, reaching approximately 32 million tons in 2010 worldwide, representing 800 million tons of CO_2_ [18]. Most agro-industrial byproducts are underused and contribute to these issues, so there is widespread interest in their valorization to mitigate them as much as possible.

Over the past few decades, great attempts have been made to develop methods to use agro-industrial byproducts, generally as animal feed or fertilizer. Still, recent reports show that developing high-value products (such as food, medicines, or cosmetics) from them is a viable alternative [19]. In this context, natural bioactive compounds are being sought to treat and prevent human diseases since they can interact efficiently with proteins, DNA, and other biological molecules to protect them against damage, thereby making them useful as natural therapeutic agents [20]. Agro-industrial byproducts have a rich and complex matrix, where various molecules present therein can interact with the consumer’s macromolecules and/or with each other, resulting in specific biological activities when consumed [21]. This knowledge, coupled with growing consumer interest in foods that provide positive health effects, makes agro-industrial byproducts, and those specifically from fruits, a particularly interesting source from which to obtain bioactive molecules that can be added to some foods. This can maximize the integral utilization of food and can be done in a viable manner. 

As globalization increases, the availability of fresh fruits in certain regions can be low or null; thus, the food industry processes significant amounts of them to reach farther corners of the world to meet local demand. Another fraction is transformed into fruit-based products, such as juices, sauces, jams, ice cream, and various others. These processes lead to the generation of agro-industrial byproducts [14], which, in the case of some fruits, can be up to 60% of their initial weight [22]. The constantly increasing demand for fruits, vegetables, and products obtained from them is accompanied by a significant amount of byproducts; however, if treated and managed properly, they can become important sources of bioactive compounds [19].

The term “bioactive compounds” refers to a diverse set of molecules capable of exerting a desirable effect on a cell, organ, tissue, system, or organism. They can be used to produce functional foods, nutraceuticals, and food additives and can be obtained from these sources. They can also be found in unprocessed foods, such as fruits and vegetables, which are the simplest form of functional foods [16]. Some bioactive compounds retain their ability to induce health-promoting activities in vitro and in vivo when extracted from their natural source and incorporated into other products. For example, phenolic compounds obtained from fruits and vegetables are notable for their antioxidant activity, making them effective agents for the prevention of oxidation-mediated processes when incorporated into functional foods or nutraceuticals [19], and are therefore commonly used for this purpose [16].

The chemical composition of agro-industrial byproducts is diverse, but those generated after the extraction of juice, oil, starch, and sugar have been particularly highlighted by other authors [14]. Dietary fiber, proteins, lipids, phenolic compounds, carotenoids, sterols, tocopherols, and terpenes are some of the most significant molecules that can be extracted from agro-industrial byproducts. These molecules have shown significant bioactivities in various models, such as antioxidants, modulators of the digestive process, and others [16,22]. Their incorporation into a food product can add value to it by inducing bioactivity, such as a higher antioxidant capacity, or improving the quality of the product, such as extending its shelf life [14,16].

## 3. Selected Bioactive Compounds Present in Agro-Industrial Fruit Byproducts

Bioactive compounds play a role in the health and maintenance of general well-being through mechanisms such as improving immunity and, therefore, the prevention and treatment of specific diseases [23]. They can have various roles in this process and can be used in different forms, for example, as antioxidants, anti-inflammatory agents, etc. There is a growing trend in the food industry toward developing and manufacturing functional and nutraceutical products [24]. This class of food products has received a lot of attention in the market due to the increased consumer interest in so-called healthy foods; thus, there is significant interest in obtaining new natural bioactive components for their development [12]. Some of the most studied bioactive compounds that can be used to increase the health benefits of a food product and which can be easily obtained from agro-industrial fruit byproducts (among others) include fiber and phenolic compounds, which are briefly discussed in this section. It should be noted that other molecules can also be obtained from byproducts, such as proteins or lipids, but they tend to be used on the main product produced from the fruit and are, therefore, less abundant in byproducts [3,25]. Some micronutrients such as carotenoids, sterols, tocopherols, terpenes, and others may also be present, but the authors of the original works do not always consider them. Moreover, due to the nature of the tissues that comprise byproducts (mainly peels and seeds and unused pulp in minor proportions) and the processes from which they were generated (processing into juice, jams, or others), fiber and phenolics are particularly abundant therein and are, therefore, the ones that commonly receive the most attention in the literature considered in the present work. 

### 3.1. Fiber

Fiber, also known as non-starch polysaccharides, is consumed as non-energy-yielding molecules. It reaches the colon almost intact, as the human upper digestive tract does not have the proper enzymes to break it down [26]. It can be classified into two broad groups, soluble (e.g., pectin) and insoluble (mainly cellulose). Soluble fiber has beneficial functions in some physiological processes; for example, it modulates the rate of release and absorption of nutrients along the gastrointestinal tract, and it is fermented by the colonic microflora, which helps maintain a healthy bacterial population. It can bind to cholesterol and bile salts in the small intestine, which promotes their increased fecal excretion, thereby reducing the concentration of serum cholesterol when regularly consumed [27]. In contrast, insoluble fiber has fewer physiological effects on the upper gastrointestinal tract and is fermented to a lesser extent by the colonic microflora; however, it plays an important role in intestinal regulation through stimulating mechanical peristalsis [26].

Both kinds of fiber have important functional properties as food additives, for example, due to their effects on binding flavor components, water retention capacity, swelling capacity, increased viscosity, and gel formation, which can be crucial when formulating certain food products [28]. The composition of dietary fiber obtained from agroindustry byproducts has been reported, some of which stand out, for example, date pulp (92.4 ± 2.7 g/100 g dw), apple pomace (89.8 ± 0.2 g/100 g dw) [29], defatted grapefruit seed (89.6 g/100 g dw) [30], lemon pulp bagasse (81.7 ± 4.6 g/100 g dw) [31], mango peel and pulp (70.0 ± 0.1 g/100 g dw), pineapple peel (75.8 ± 0.2 g/100 g dw) [32], and pomegranate pulp (56.3 ± 0.4 g/100 g dw) [33]. These fiber-rich byproducts may be useful as ingredients when designing products whose dietary fiber should be increased or for specific applications in the manufacture of various foods.

### 3.2. Phenolic Compounds

Phenolic compounds include a wide range of amphipathic substances (>8000 different known molecules), which are synthesized mainly through the shikimic acid, pentose phosphate, and phenylpropanoid pathways. More structurally complex phenolics are referred to as polyphenols, although they all have in common at least one aromatic ring with one or more hydroxyl substituents [34]. These compounds are most commonly known because of their antioxidant properties since they can inhibit or delay oxidation by reducing the concentration of transition metal ions (mainly iron and copper) and free radicals [28]. Free radicals, in particular, can be effectively neutralized by phenolic antioxidants; they donate an electron or hydrogen atom to them, paring their unpaired electrons and reducing their reactivity [35]. Once a phenolic compound neutralizes a free radical, its cell-damaging effects are minimized since they can no longer oxidize macromolecules relevant to biological systems. These simple, but highly effective protective actions, have been associated with reducing the risk of developing multiple pathologies, such as cardiovascular disease, cancer, diabetes, and age-associated degenerative conditions [36].

In the food industry, antioxidants are also used to preserve flavor and color by preventing the oxidation of their constituents [37]. They can also be added to foods to balance the free radical production rates and release once consumed, thus acting as exogenous antioxidants [38]. Some of the main advantages of using industrial byproducts as a source of phenolic compounds are that they are a low-cost, readily available source [39], making their valorization of utmost importance for promoting human health, decreasing cost, and minimizing waste. Some agro-industrial byproducts particularly notable for their high phenolic content are apple pomace, blackcurrant, cranberry peel, cherry, cactus peel, mango peel, and grape peel, among others [40]. These and other byproducts can be used to exert various biological activities, where an antioxidant property is desirable. They have been considered of particular interest to preserve or fortify food products, where they can be used whole or further processed to isolate and concentrate their phenolic compounds.

Obtaining bioactive compounds such as fiber and phenolic compounds from agro-industrial fruit byproducts is a promising strategy to reintroduce them into the food processing chain. This can contribute to making products with significant physiological functions that benefit the consumer’s health. In this sense, adding agro-industrial fruit byproducts or bioactive compounds extracted from them can modify the composition and functionality of foods, improving both their applicability and quality.

## 4. Selected Foods Enriched with Agro-Industrial Fruit Byproducts, and Their Beneficial Health Effects

**Figure 1:**
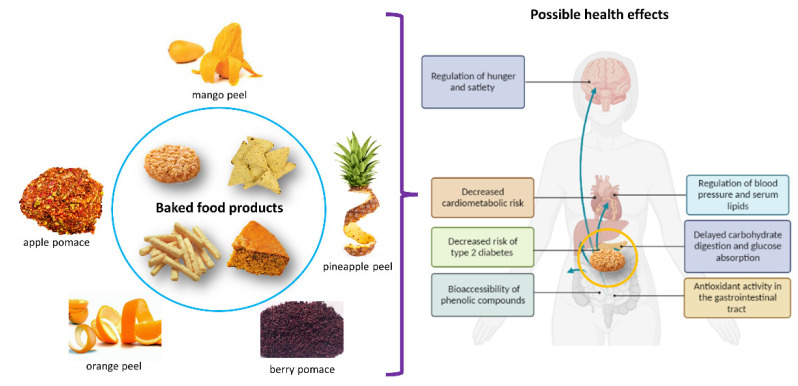
Examples of agro-industrial fruit byproducts that have been used to supplement baked food products (biscuits/cookies, corn chips, extruded snacks, and cakes) and some possible health effects that these can induce on the consumer.

Agro-industrial fruit byproducts have been used to enrich various foodstuffs. Their addition to baked products has been explored because they are optimal candidates to be supplemented since they are highly consumed worldwide and are commonly made with refined flours and ultra-processed ingredients, making them high glycemic index foods. Therefore, this section focuses on the most relevant findings of using agro-industrial fruit byproducts for this purpose. It also discusses the resulting physicochemical characteristics, sensory properties, and health effects of these products. The studies mentioned in the main text are summarized in Table 1 and Figure 1. 

It should also be noted that byproducts from the fruit industry have also been used to improve foods of animal origin; for example, eggs, where hens fed with a diet supplemented with sea buckthorn had a significant increase in n-3 fatty acids and antioxidant capacity, as well as positive effects on other health-related indices. Dehydrated pineapple byproducts have been added to improve the properties of meat products (pH, hardness, and color), which was attributed to their bromelain content. Likewise, they have also been used to fortify spreadable cheese, where it was observed that, by adding red and white grape pomace, its content of phenolic compounds and antioxidant capacity increased significantly [56,57,58]. However, the strategy for these foods differs from those considered in the present work.

### 4.1. Cookies/Biscuits

Approximately 10 million tons of apple pomace are obtained each year during juice production worldwide, most of which is not commonly used. Apple pomace comprises pulp, peels, seeds, and stems, making it rich in fiber and phenolic compounds [59]. Alongi, Melchior, and Anese [41] produced Golden Delicious apple pomace flour obtained on a laboratory scale and used it to partially substitute wheat flour (10 and 20%), with which reduced glycemic index cookies were made (glycemic index describes changes to the consumer’s serum glucose in response to ingesting food or beverage, and can be significantly lowered by, among other factors, a higher fiber content due to its effect on decreasing its intestinal absorption). The apple pomace's total phenolic compound concentration was 1.1 mg GAE/g dw, and fiber was 36.6%. As for its physicochemical properties, it was reported that, by increasing the percentage of apple pomace flour, the cookies' thickness and firmness decreased compared to the controls. This may have been due to interactions within the food matrix, such as changes to its water absorption capacity exerted by the pomace fiber or the partial decrease in wheat flour that reduced gluten content, limiting the formation of interaction within the matrix. The authors performed a sensory evaluation, where the panelists reported that the samples with 20% apple pomace had a stronger fruit flavor, in contrast to the samples with 10%, which were perceived similarly to the control. They also performed an in vitro digestion to evaluate their glycemic index. They reported a maximum glucose concentration of 98 and 97 mg/g dw for the 10 and 20% samples, respectively, values significantly lower than those of the control (120 mg/g dw). Other authors have reported changes in sensory perception of baked products when a higher concentration of apple pomace is used [46]. The control was therefore considered a high glycemic index food (70), while samples added with 10% (65.7) and 20% (60.8) apple pomace could be considered of intermediate glycemic index, which the authors attributed to their pomace’s fiber content. The authors then used apple pomace derived from industrial processing to corroborate their results, which contained a high fiber content (47.2%). It was also processed into flour and used to replace wheat flour (20%), yielding a product with an in vitro glycemic index of 62.0, similar to those produced using laboratory-obtained pomace. Based on their results, the authors propose that the industrial by-product could be used as a functional ingredient to lower the glycemic index of wheat cookies.

This data regarding the use of apple pomace is promising, although a proximate analysis and content of phenolic compounds of the substituted cookies were not reported. It has been shown that bioactive compounds, such as phenolic compounds, can modulate the digestive process by inhibiting key enzymes and the rate of intestinal transit, which slows down carbohydrate digestion and glucose absorption, thereby decreasing a product’s glycemic index [60]. Thus, data about the cookies’ phenolic composition may be of value to establish an effective dose of phenolics needed to obtain a decrease in their glycemic index. Apple byproducts (its peel in particular) are rich in phenolic compounds such as chlorogenic acid, procyanidin B2, and epicatechin, inhibiting α-amylase and α-glucosidase, a key effect in the reduction of postprandial glycemia. The fiber present in this and other byproducts can also reduce its glycemic index through additional mechanisms. For example, soluble fiber can increase the viscosity of the matrix at the gastrointestinal level, contributing to the formation of a gel. The latter can wrap starch grains, protecting them from the amylolytic activity of digestive enzymes, thereby slowing down the release of free glucose [61]. Although insoluble fiber does not directly influence postprandial glucose fluctuations, it does influence glycemic response, as it has been shown to alter intestinal transit time, which is associated with a significant reduction in the risk of type 2 diabetes [62]. It should also be noted that the gelatinization of starch occurs by baking the product. Consequently, it cannot be hydrolyzed since it becomes inaccessible to digestive enzymes in the gastrointestinal tract, so it does not contribute to the glycemic response [61]. These studies are particularly promising for formulating foods for managing type 2 diabetes since patients may benefit from consuming products rich in phenolic compounds and dietary fiber.

Pomegranate peels are another agro-industrial byproduct rich in fiber and phenolic compounds such as ellagitannins, gallic acid, ellagic acid, and their derivatives. Colantuono, Ferracane, and Vitaglione [42] supplemented cookies with pomegranate peel (7.5%) and evaluated the bioavailability and ability of their phenolic compounds to exert antioxidant activity throughout the gastrointestinal tract and modulate digestive enzymes in vitro. The data showed a potential bioavailability of phenolic compounds and an increased antioxidant activity due to their presence. In addition, compounds released during the duodenal phase exhibited inhibitory actions on α-amylase, α-glucosidase, and lipase. These findings demonstrated that adding a relatively low percentage of pomegranate peel to a baked product increased the bioaccessibility of phenolic compounds in the duodenum, with consequent antioxidant protection and modulation of the digestive process.

Phenolic compounds can be used as a leading indicator of some bioactive properties of food, including inhibition of glucose-releasing enzymes [63]. The previously discussed findings of Colantuono, Ferracane, and Vitaglione [42] may be attributed to pomegranate peel's high anthocyanin and tannin content. Punicalagin, ellagic acid, and gallic acid are some of the most relevant for this process since they can significantly inhibit these enzymes [64,65]. Starch is the most common complex carbohydrate in food, and its digestion in the gastrointestinal tract is primarily mediated by α-amylase and α-glucosidase. The rate of glucose release and absorption along the gastrointestinal tract also contributes to the regulation of the physiological mechanisms underlying hunger and satiety [42,63], these results suggest that the phenolic compounds present in pomegranate peel could have a positive impact on consumer satiety, but additional data is required to conclusively validate it on consumers. In addition to its possible effects on health, it may be adequate to complement with physicochemical and sensory analyses, which could clearly determine the impact of the byproduct on variables that could alter its original properties.

Coffee is one of the most commercialized products worldwide; one of the most relevant species is *Coffea arabica*, which comprises 70% of its total production. To produce coffee, the berries are processed by wet methods that generate large amounts of byproducts (pulp) since, for every 2 tons of processed berries, 1 ton ends up as pulp [66]. Moreno, Cozzano, Pérez, Arcia, and Curutchet [43] used 10% dried coffee pulp obtained from the wet processing of *Coffea arabica* as a source of antioxidant fiber to develop salty biscuits with high fiber content. Its proximate composition confirmed an increase in fiber, making it a “high in fiber” product. The samples' total phenolic content and antioxidant capacity were quantified in addition to performing an in vitro digestion; however, no physicochemical changes to the product were analyzed. The phenolic content of supplemented biscuits and their antioxidant capacity (1168.58 ± 23.50 µmol TE/30 g) were significantly higher than those of the controls (approximately 250 µmol TE/30 g), a pattern that persisted after the in vitro digestion was performed. A sensory evaluation was also carried out, where consumers rated the taste of the supplemented samples as satisfactory since there were no significant differences with the controls. The authors also divided their panelists into informed and non-informed groups and observed that knowing about their health-related properties (rich in fiber and antioxidants) generated a positive impact on their opinion. The authors, therefore, proposed that the formulation of biscuits with 10% dry coffee pulp was successful, according to their sensory acceptability and potentially functional properties.

The specific profile of bioactive compounds present in the samples developed by Moreno, Cozzano, Pérez, Arcia, and Curutchet [43] was not reported, nor were the potential health effects of the samples; however, coffee pulp has been analyzed by others, and has been shown to contain numerous highly bioactive substances, such as chlorogenic acid, cyanidins, tannins, carbohydrates, soluble fibers, proteins, and minerals [67]. These can exert antioxidant properties and protect against some chronic diseases, such as some types of cancer and metabolic syndrome, by diverse mechanisms such as normalizing blood pressure, glycemia, and serum lipids. This makes coffee pulp-supplemented products of potential interest to prevent or mitigate these conditions [64]. The bioactive effects of coffee byproducts have been studied, albeit, not as part of an edible product. For example, Magoni et al. [68] observed that coffee byproducts inhibit the release of IL-8 in human gastric epithelial cells. This chemokine is considered the main responsible for the development of human gastritis and is a target for the search for new compounds to treat it [69]. Consumption of 2 mg of coffee pulp extracts, which is considered a low amount, leads to a potential gastric concentration of 100 μg/mL, a threshold that showed biological activity under experimental conditions. Phenolic compounds in coffee pulp show anti-inflammatory effects in a cellular model of gastric inflammation [70], making this class of compounds a potential candidate for explaining the anti-inflammatory activity observed in this study. This evidence complements what is described by Moreno, Cozzano, Pérez, Arcia, and Curutchet [43] and allows to propose coffee byproducts as a source of compounds with gastrointestinal bioactivity, although further studies are required.

Grapes are one of the most cultivated and consumed fruits around the world. Interest in their consumption has increased considerably, partly due to their rich antioxidant effects and dietary fiber content [71,72]. Most grapes are cultivated to produce wine, a process that generates about 20% of pomace, composed of stems, peels, seeds, and pulp [73]. It has been reported that grape pomace is rich in phenolic compounds and of high antioxidant activity, making it a potential ingredient for functional foods [74]. Theagarajan, Narayanaswamy, Dutta, Moses, and Chinnaswamy [44] developed grape pomace-supplemented biscuits (2, 4, 6, and 8%) and observed a significant increase in their contents of protein (5.13, 5.25, 5.69, and 6.44%, respectively) and fiber (2.30, 2.59, 2.62, and 3.04%, respectively), as compared to the controls (3.10% and 1.71%, respectively). The authors reported that the grape pomace used had a total phenolic content of 280.63 mg GAE/g dw and antioxidant activity of 57 ± 2 μg/mL (DPPH method, IC_50_), while anthocyanins were the most noteworthy compounds (488.80 ± 5.80 mg/100 g). As for their physicochemical properties, it was observed that the cookies became darker than the controls as the pomace content increased, while no effects were documented on their shapes or their water activity. Regarding their sensory analysis, samples supplemented with 4% and 6% had good acceptability; however, as the percentage of grape pomace increased, its acceptance decreased due to the bitter taste of the pomace since the panelists generally indicated that the cookies had a pomace flavor. Other authors have also observed that percentages greater than 15% of grape pomace can decrease sensory acceptance in similar products [75]. The content of phenolic compounds of the samples supplemented with 6% pomace increased (4.03 mg GAE/g), while those with 4% had similar values to the controls (3.41 mg GAE/g). The antioxidant activity of the samples with 4% pomace was 0.85 ± 0.4 mg/mL of extract, while those with 6% had a value of 0.72 ± 0.5 mg/mL of extract, and a total anthocyanin content of 102 mg/100 g and 173 mg/100 g, respectively. Thus, it is apparent that the antioxidant activities were not directly correlated with the content of phenolic compounds or anthocyanins. Some physicochemical interactions may have been responsible for this phenomenon, although they were not specifically studied.

The health effects of the grape pomace-supplemented samples developed by Theagarajan, Narayanaswamy, Dutta, Moses, and Chinnaswamy [44] were not evaluated, although other authors have analyzed the effects of consuming similar byproducts. For example, Rivera et al. [76] supplemented the atherogenic diet of mice with ischemic heart disease (7 or 14 days) with grape pomace extracts and observed a decrease in premature death. This can be attributed to various mechanisms exerted by molecules present in the byproduct, which delay the progression of atherosclerosis and coronary heart disease and improve cardiovascular health in general. α-tocopherol and some phenolics (gallic acid, catechin, flavonols, and malvidin, among others) are the main antioxidants present in grape pomace, which could have modulated lipid peroxidation to contribute to the observed effect in mice [77]. Therefore, grape pomace is an excellent source of bioactive compounds that can be used to develop biscuits. However, the health effects of consuming the supplemented product should be evaluated, particularly in humans.

Prickly pear is obtained from cacti, which are highly cultivated in Mexico. Its pulp is commonly consumed fresh, but is also used to manufacture juices, alcoholic beverages, and baked products [78]. Its seeds are used to extract its oil, leaving the peel as an unused byproduct representing approximately 30–60% of its total weight [8]. It has been reported that the peel has a high content of bioactive compounds, as well as dietary fiber and protein, making it an ingredient of interest for the preparation of baked products. Bouazizi, Montevecchi, Antonelli, and Hamdi [45] evaluated the incorporation of prickly pear flour with wheat flour to prepare biscuits and determined the resulting products' physicochemical properties and sensory characteristics. The peels were dried in an electrothermal oven at 45 °C for 48 h, processed into flour, and used in 3 formulations (10, 20, and 30%). The content of phenolic compounds increased in each formulation (360, 477, and 575 mg GAE/100 g dw, respectively), as did that of fiber (2.12, 2.45, and 3.14%, respectively), carotenoids and betalains (1.77/49.7, 2.57/118.7, and 3.54/176.7 mg/100 g dw, respectively). As for their physicochemical properties, water and oil retention capacity increased as the percentage of prickly pear flour increased, which was attributed to its high content of hydrophilic components such as soluble fiber. The hardness of the dough used was evaluated, where an increase was observed in the cookies supplemented with byproducts at 20%. There were no differences in cohesiveness between the samples. This finding was also associated with the high fiber content and its hydroxyl groups, which allows the formation of strong interactions between gluten proteins (hydrogen bonds). A sensory evaluation showed that the scores for color, smell, and taste increased, particularly in those supplemented with 20% prickly pear peel flour, as compared to the control; the parameters of acceptance increased mainly in samples with higher percentages of the byproduct, which was attributed to some reducing sugars present in prickly pear flour that may have enhanced their aroma. The partial use of prickly pear flour to produce baked products is a promising strategy to increase their fiber and bioactive compound content and their antioxidant activity. It can be readily obtained from agro-industrial processing, which currently has no significant application and is discarded. Prickly pear peel is considered a rich source of dietary fiber, vitamin C, and betalains [15]. These compounds are associated with beneficial health effects such as cardiovascular disease, diabetes, cancer, and hypertension, among others [79]; however, more studies are required since there are still few in which prickly pear byproducts are used.

Pineapple, apple, and melon are consumed and processed extensively to produce juices and pulps, leaving large amounts of byproducts mainly intended for animal feed. They have considerable amounts of fiber, minerals, vitamins, and phenolic compounds [80]; thus, de Toledo, Nunes, da Silva, Spoto, and Canniatti-Brazaca [46] used byproducts of pineapple (central axis), apple (endocarp), and melon (husks) generated during industrial processing, which were freeze-dried to obtain different flours. Flour substitution was performed at 5, 10, and 15% to supplement cookies; the authors report that they performed preliminary tests with additions of 17, 18, and 20% of byproducts, however, the samples had undesirable characteristics in terms of dough malleability. As for their physicochemical properties, a variation in their diameter was observed in samples where pineapple flour was used, which was attributed to the action of bromelain, which is generally used in baked products. It has been shown that adding high levels of byproducts may sometimes be technologically unacceptable (as previously mentioned), or regarding the sensory parameters of the final product (strong bitter aftertaste) [81]. The authors reported that cookies with pineapple flour had increased soluble and insoluble fiber values at 5% (1.30 and 1.14 g/100 g), 10% (1.68 and 1.17 g/100 g), and 15% (2.08 and 1.05 g/100 g), apple byproducts at 5% (1.23 and 1.61 g/100 g), 10% (1.96 and 1.45 g/100 g), and 15% (2.52 and 1.64 g/100 g), and melon byproducts at 5% (2.90 and 1.77 g/100 g), 10% (2.73 and 2.31 g/100 g), and 15% (3.54 and 2.92 g/100 g). The flour substitution imparted a darker color to the samples. However, a sensory analysis showed that adding the highest percentage (15%) still had good overall acceptability, but this was fruit-dependent. Specifically, samples with 15% pineapple flour had the highest acceptability, followed by samples with 15% apple flour, the control, and samples with 15% melon flour. The lower acceptability of the melon samples can be related to the acidity and bitter taste of the peels used to produce the flour since the authors stated that its addition did not influence the appearance or aroma of the samples. As for the purchase intention of the supplemented products, the panelists stated that they would buy at least some of them. This study further reaffirms that higher percentages of byproducts tend to decrease the sensory acceptance of the final product since they may impart a slightly bitter and acidic taste, as was the case in those discussed herein. Such flavors are related to the high content of phenolic compounds, which may not be as palatable at higher concentrations even if they exert important health properties. These byproducts are rich in bioactive compounds, although the authors did not report the profile of phenolic compounds or antioxidant capacity, which would be useful to gain further insight into the potential health effects of the developed samples. As mentioned, the fiber content was evaluated and found to have been increased; fiber consumption is known to be linked with a reduced risk of chronic diseases such as obesity, cardiovascular disease, and diabetes [82]; thus, increasing it can be considered a desirable effect.

The bioactive content of blackberries is well-known, with phenolic acids, flavonoids, and tannins of high antioxidant capacity being some of the most relevant molecules present in them [47]. Fresh berries can induce a strong astringent sensation; thus, they are mainly used to produce juices, jams, or other goods, leading to the generation of large amounts of byproducts, with approximately 20% of the fruit ending up as pomace [83]. Gagneten, Archaina, Salas, Leiva, Salvatori, and Schebor [50] developed gluten-free chocolate cookies supplemented with blackberry pomace. The byproduct had a significant content of dietary fiber (32.3%), protein (4.2%), total phenolic compounds (37.5 mg GAE/g dw), anthocyanins 18.0 (mg C3G/g dw), and antioxidant activity (22.1 mg GAE/g dw, TEAC method). As for the physicochemical properties of the cookies, an intense red color was observed due to the high content of anthocyanins in the byproducts. In terms of water content, there were no significant differences between the supplemented samples and controls, but their water retention capacity increased in supplemented samples. This was attributed to the presence of fiber and low molecular weight compounds, which could promote interactions within the matrix and water molecules. The authors made an internal sensory evaluation to decide on the final formulation of 3.75% blackberry powder because higher percentages unacceptably compromised the products’ organoleptic properties; they specifically mention an unpleasant aroma and astringent flavor. The other percentages of addition considered in the study were not reported. It is noteworthy that, regarding smell and color, most consumers reported both formulations as ideal, while most accepted the flavor of both samples. The supplemented cookie showed a higher dietary fiber content (4.8%) as compared to the control (1.9%), making it possible to classify it as a source of dietary fiber since its content was ≥4.2% [84]. It also had a 62% higher content of phenolic compounds (2.5 mg GAE/g dw) compared to the control. An in vitro digestion revealed that more than 50% of the digested sample had a high antioxidant capacity that could reach the large intestine and induce an antioxidant environment that could potentially preserve gastrointestinal health. Thus, blackberry pomace-supplemented cookies may provide important bioactives that can improve health, according to their higher fiber and bioactive content, in addition to their antioxidant capacity.

According to the data above, various agro-industrial fruit byproducts have been used to supplement cookies/biscuits. They were mostly produced at a laboratory scale to partially replace wheat flour, the main ingredient used to make them. It should also be noted that most studies do not report optimization data or how the addition percentage was selected. Because they were produced in the laboratory, they were not pretreated before using them; this is necessary for industrially-sourced material for quality and safety purposes. The precise composition of the byproducts used (bioactive compounds) is also not always reported, nor are any potential bioactivities in vitro or in vivo.

### 4.2. Corn Chips

Although mango is mainly consumed fresh, various derived products such as juices, nectar, and purées are available. Consequently, to its industrial processing, mango byproducts like peel and seed are discarded, which can constitute between 35 and 60% of its weight but have been shown to contain a high concentration of bioactive compounds [85]. Zepeda-Ruiz, Dominguez-Avila, Ayala-Zavala, Robles-Sanchez, Salazar-Lopez, Lopez-Diaz, and Gonzalez-Aguilar [48] reported that the addition of 10 and 15% mango peel to baked corn chips resulted in a product with the highest overall acceptability and, in particular, their smell, color, flavor, and texture were highly evaluated, as compared to the control and samples supplemented with 20% mango byproducts. Consumers reported that the products mostly had an acceptable smell and taste, but those with the highest percentage of mango peel had a slightly bitter taste, which is characteristic of the phenolic compounds present in them (tannins). Their color was also appreciated since consumers liked the golden hue imparted by the peel. As for their physicochemical properties, a texture analysis revealed changes to their hardness in response to adding mango peel, which the authors argue may have impacted their acceptability. The content of phenolic compounds and antioxidant activity of the supplemented products was up to ten times higher than the controls. An in vitro digestion revealed a decrease in dialyzable glucose after the intestinal stage; thus, the authors proposed that mango peel can be used to produce baked corn chips with good sensory acceptability, antioxidant properties, and a lower glycemic index. The effects of mango peel on the samples’ potential health properties may be due to its phytochemical profile, such as fiber, phenolic compounds, carotenoids, tocopherols, and ascorbic acid, among others, which have favorable effects on glucose homeostasis. In particular, phenolic compounds can inhibit α-amylase and α-glucosidase, key enzymes in regulating intestinal glucose absorption and systemic homeostasis. Simultaneous intake of dietary fiber and these bioactive compounds has been reported to prevent some types of cancer, cardiovascular disease, and other non-communicable diseases [86]. Mango peel could therefore be used as a functional ingredient to decrease the glycemic index of the product, as well as other properties due to its high content of bioactive compounds.

Others have also considered the formulation of healthier corn chips with the addition of agro-industrial fruit byproducts because these snacks are commonly high in carbohydrates, fats, and glycemic index. Mayo-Mayo, Navarrete-Garcia, Maldonado-Astudillo, Jimenez-Hernandez, Santiago-Ramos, Arambula-Villa, Alvarez-Fitz, Ramirez, and Salazar [49] developed corn chips supplemented with mango peel or roselle byproducts (separately), both of which have been recognized for their high content of fiber and antioxidant compounds. They produced four samples; two supplemented with mango peel (5 and 10%) and two more with roselle byproducts (5 and 10%). The samples with the highest fiber content were those added with 10% mango peel (32.38 ± 1.16%), followed by those with 10% of roselle (29.30 ± 1.20%), values that were significantly higher than the controls (8.81 ± 0.30%). The authors mention that before frying their products, they used a dehydration method that reduced the oil content by 58%, which they propose as an alternative method to produce low-fat chips. The samples with the highest content of phenolic compounds were those added with 10% mango peel (3.73 ± 0.14 mg/100 g dw), followed by those supplemented with 10% of roselle byproducts (1.56 ± 0.09 mg/100 g dw), with values that were approximately five and two times more than the controls, respectively (0.76 ± 0.01 mg/100 g dw). However, there were no significant changes in antioxidant capacity, with the caveat that it was only determined by a single method. The glycemic index of the supplemented products decreased significantly in those with 10% mango peel (68.25 ± 1.21) and 10% roselle byproducts (69.02 ± 3.37), as compared to the controls (84.41 ± 3.17). The physicochemical properties of the supplemented products revealed a decreased hardness, which the authors attributed to gelatinized pores in the starch matrix present on all samples before cooking. Furthermore, the soluble fiber content can contribute to forming a cross-linked network that can solidify the starch matrix, avoiding the creation of pores. As for the addition of roselle calyces, their insoluble fiber favored the weakening and collapse of the starch matrix and the creation of pores, which also contributed to the aforementioned reduced hardness. A sensory evaluation showed no significant differences in smell, texture, and color parameters, although chips with 5% mango peel and 10% roselle byproducts had the highest acceptability. Furthermore, the panelists commented that the flavor changes caused by adding mango peel improved their acceptability. Based on their evidence, the authors concluded that up to 10% of mango or roselle byproducts could be added without affecting their sensory acceptance. Consumption of roselle byproducts can potentially exert beneficial health effects, as evidenced by both in vivo and in vitro studies, particularly because of its rich fiber content (36–38%) and phenolic compounds (3–4%) [87]. Amaya-Cruz et al. [88] studied the effect of a 4% roselle byproducts supplementation in a hypercaloric diet used to feed rats, which yielded a reduction in body weight (−10%), adipocyte hypertrophy (−17%), insulin resistance (−48%), and hepatic steatosis (−15%). Thus, roselle byproducts can exert potential effects on weight control and improve insulin resistance and lipid metabolism. However, their effectiveness when used to supplement edible products remains to be studied.

Corn-based products, such as corn chips, are usually low in protein and simple carbohydrates, which contributes to their high glycemic and caloric index [10], a finding that is congruent with the data reported in the studies discussed in this section. These studies suggest that adding flour from byproducts could improve the glycemic index due to the increased fiber and phenolic content, although this should be studied further in vivo. Their nutritional profile can also be improved according to a considerable increase in their bioactive compound content and antioxidant activity, which can positively affect health. Maintaining their sensory attributes to make them desirable to the consumer is also required, although some works do not report sensory analyses. Byproducts used were not pretreated, nor is it reported how the authors selected the percentages of supplementation; thus, there is still potential to improve the quality of the final product and its acceptability.

### 4.3. Extruded Snacks & Breadsticks

Blackcurrant is one of the most used fruits in the juice industry, whose processing generates a large number of byproducts, namely, skin, seed, and stems, which are rich in bioactive compounds such as anthocyanidins, fiber, γ-linolenic acid, α-linolenic acid, and stearidonic acid, which could have beneficial effects on health [89]. These byproducts have been used mainly as animal feed or are simply incinerated, so other alternatives have been sought for their use, but they can be used to produce nutritious snacks to supply their growing demand [90]. Mäkilä et al. [91] produced an extruded product from blackcurrant press residues obtained from its industrial juice processing. One batch of these residues received an enzyme pretreatment, and the other batch a non-enzymatic one. Both batches were dried at 40 °C to obtain flour that was then used to prepare 6 extruded formulations. These consisted of 27–28% blackcurrant byproducts, 38–39% cereals (barley flour, oatmeal, or oat bran), and 14–15% potato fecula, as well as sugar (4.7–4.8) and salt (0.5%); extrusion formulations and parameters were selected based on preliminary tests. As for their physicochemical properties, all extruded samples presented color changes, which could be the result of the Maillard browning reaction that can occur due to the sugars and amino acids present in the sample, and the high temperatures of the barrel. Changes in hardness, expansion, and porosity were also recorded; these may have been due to interactions between the flour and blackcurrant pomace since the carbohydrates and lipids in the dough can lead to the formation of amylose–lipid complexes, which then prevent starch gelatinization and lead to a decreased expansion in extrusion.

Regarding their proximate composition, extrudates based on enzymatic-treated residues had a higher energy content, protein, fat, and fiber due to the enzyme-assisted pressing that efficiently decomposed the structure of the berry. It was observed that the extrudate produced from non-enzymatic material and barley or oat flour had a greater expansion, lower hardness, pH, and higher content of fructose, glucose, and fruit acids, which positively contributed to their sensory acceptability. Specifically, their sensory acceptability showed that barley samples with non-enzymatically-treated blackcurrant byproducts had the highest score, while enzymatically-treated oatmeal extrudates had the lowest. Authors also observed that the female panelists gave lower ratings than men, which differs from other studies where women show greater acceptability towards healthy products than men [92]. The physicochemical modifications that the products had in response to the addition of blackcurrant byproducts should be documented in detail since a product’s quality may be affected. The identification and quantification of the bioactive compounds and antioxidant activity should also be considered since these are commonly associated with some positive effects on the consumer’s health, as well as validating these bioactivities in vitro or in vivo. Other studies have reported that blackcurrant byproducts have high concentrations of anthocyanins and flavonols, which, when used to supplement white rabbits’ diet, a decrease in the concentration of putrefactive metabolites and the activity of β-glucuronidase in the digestion of the intestine were observed, as well as modulation of their serum lipids (triglycerides, total cholesterol, and HDL) and an increase in antioxidant capacity [93]. This partly supports the use of these byproducts as a health-promoting ingredient, although the supplement product's health effects should still be analyzed.

Due to the growing interest in byproducts and their high content of bioactive compounds, they have been used to develop nutritious snacks. For example, mango pulp and papaya are widely processed into juices, nectars, porridges, jams, etc., leaving high volumes of unused peel. These have been reported to contain a higher concentration of phenolic compounds than the pulp [94]. Fontes-Zepeda, Dominguez-Avila, Lopez-Martinez, Cruz-Valenzuela, Robles-Sanchez, Salazar-Lopez, Ramirez-Wong, Lopez-Diaz, Pareek, Villegas-Ochoa, and Gonzalez-Aguilar [51] supplemented corn-based extrudates with mango and papaya peel (15%), which were obtained under laboratory conditions; the authors did not report how they decided on the supplementation percentage. Higher content of phenolic compounds was found in the extrudates with mango peel, while those with papaya peel had a higher carotenoid content. Regarding their physicochemical properties, the extrudate with papaya peel had greater changes in terms of expansion and proximate composition (proteins and lipids), while both extrudates had a lower fracturability than the control. This may have been due to interactions between particle size, temperature, and screw speed. Likewise, color changes observed in response to the addition of mango and papaya peel imparted a darker color to the samples, which was attributed to the presence of carotenoids, as well as reducing sugars. The samples with greater mango peel content had the highest sensory acceptability, suggesting that the panelists prioritized taste more than appearance or texture. An in vitro analysis was also carried out, where a greater release and absorption of bioactive compounds and antioxidant capacity was observed in supplemented samples, which has been previously reported to exert potential health effects [95]. 

Breadsticks are another type of baked product that has been supplemented with byproducts and which are widely consumed around the world. The wine industry generates a large amount of waste (grape pomace) that can be a potential source of bioactive compounds (phenolic acids and dietary fiber) [96] with various reported health effects [97]; thus, their use as ingredients in baked foods could increase their nutritional properties. Rainero, Bianchi, Rizzi, Cervini, Giuberti, and Simonato [52] added grape pomace (5 and 10%) to breadsticks and evaluated their properties. As for the rheological properties of the dough, changes were found in response to the presence of grape pomace, such as increased water absorption. The authors attributed this effect to the fiber content of the pomace, which favors water retention in the food matrix, according to the presence of numerous hydroxyl groups that can form hydrogen bonds [45]. It was also observed that some rheological properties decreased when adding the grape pomace, findings that can be related to the protein content of wheat flour that helps the development of the gluten network. Samples with 10% pomace had a high fiber content that could allow them to be classified as “high in fiber,” potentially helping the consumer reach the recommended daily intake for adults. Furthermore, their insoluble dietary fiber fraction may exert significant health effects, such as delaying gastric emptying, promoting intestinal transit, and overall digestive health [33,62]. While the content of lipids and sugars did not vary between samples, there was a significant increase in their content of phenolic compounds (72.21, 124.54, and 171.83 mg GAE/100 g, for samples with 0, 5, and 10%, respectively) and antioxidant capacity according to the FRAP (360.60, 1962.94, and 2801.00 μg TE/100 g for samples with 0, 5, and 10%, respectively) and ABTS (233.53, 701.06, and 1139.25 μg TE/100 g for samples with 0, 5, and 10%, respectively) assays. Regarding their sensory acceptability, it was similar for the samples with 5% byproduct and the control, while the one with 10% had a lower rating. The panelists reported that as the percentage of addition of byproducts increased, the samples smelled like wine. The sample added to 10% was perceived to have a remarkable acid and bitter taste in terms of flavor. Because the 5% formulation had considerable values of bioactive compounds as well as good sensory acceptability, grape pomace could be used as a functional ingredient at this percentage. The study mentioned some positive health effects of the insoluble fiber content; however, no studies were performed, so further experiments could be used to corroborate them in vitro and/or in vivo.

Studies on the production of baked foods supplemented with byproducts tend to focus mainly on their physicochemical characteristics; however, it is important to complement this basic research with other analyses, such as their bioactive compound content, their health effects, and their sensory acceptability, as well as any pretreatments before incorporating the byproducts, some which are not always considered or reported. It has been reported that consumers tend to reject products due to unexpected or uncommon organoleptic changes; thus, most of these can be supplemented with no more than 15% byproducts since higher values result in changes beyond the acceptable threshold [9]. There are studies in which consumers are provided with nutritional information about new products and their possible beneficial effects on health, where their perception of the product changed to accept these products better, even if their sensory profile may not be as pleasant [98]. Therefore, it is of utmost importance to provide a product whose criteria for preparation integrate both their nutritional properties and their sensory acceptability, thus allowing the successful development of new products.

### 4.4. Cakes & Muffins

Agro-industrial fruit byproducts have been used as economical sources of functional ingredients like antioxidants, vitamins, and fiber, among others. Their use is a significant challenge when incorporating them into some baked products, most notably cakes, since their presence may decrease the product’s perceived quality (parameters of sensory acceptability) and alter some physicochemical properties (water retention capacity, particle size, and gel formation activity among others) [9]. Some researchers have tried to optimize the percentages of byproducts that can be added to cakes, for example, apple, carrot, and orange byproducts left over from juice production, an industry that generates high volumes of unused matter (approximately 25–60% of the fruits’ weight). Kirbas, Kumcuoglu, and Tavman [53] examined the effects of using different sources of fiber (apple, carrot, and orange pomace) on rice's rheology and quality parameters flour-based cakes. The authors obtained the pomace under laboratory conditions, dried it (60–70 °C) and ground it. Different addition percentages were used (5, 10, and 15%), although it was not reported how these specific values were selected. Increased viscosity and hardness were observed, in addition to decreased volume; partially substituting rice flour increased the content of fiber, protein, ash, and sugar, which could be associated with said changes, since these components can affect the aeration process of the dough by altering its structure and releasing trapped air. The cake with 5% orange pomace had the highest sensory acceptability and higher protein and dietary fiber content; however, the authors observed that texture evaluations were significantly reduced at higher percentages. The cakes containing carrot pomace received lower scores in terms of taste, suggesting that a higher percentage of addition reduces the overall acceptability of the product. The content of phenolic compounds and antioxidant activity was not reported; however, they may likely have increased since the byproducts used are rich in phenolics with various reported beneficial effects on health. For example, carrot pomace has a good residual vitamin, mineral, and dietary fiber content, which can reduce the risk of some cardiovascular diseases and some types of cancer, which supports its current interest as an ingredient in the development of extruded products, bread, and cakes [99]. Dietary fiber and flavonoids (mainly hesperidin) present in orange pomace have also received considerable attention due to their known positive effects on the gastrointestinal tract and their potential to decrease the postprandial glycemic response in populations with cardiometabolic risk [100].

Muffins are another highly-consumed baked product worldwide due to their flavor and texture; however, they can be rich in calories and low in dietary fiber, making them optimal candidates to be supplemented with fruit byproducts [101]. Grape byproducts from the wine industry include skin, seeds, and unused pulp, which retain a high concentration of bioactive compounds [11,72]. Troilo, Difonzo, Paradiso, Pasqualone, and Caponio [54] evaluated the physicochemical, nutritional, and sensory characteristics of muffins supplemented with grape pomace flour of different particle sizes (600–425, 425–300, 300–312, and 212–150 μm). The samples’ lipid and ash contents increased in those supplemented with the pomace of finer particle size (212–150 μm). In comparison, proteins and carbohydrates were higher in samples supplemented with particles of larger size. The fiber content increased in samples with particles of 300–312 μm; all muffins with 15% grape pomace flour could be classified as a “source of fiber.”

Regarding phenolic compounds, anthocyanins, and antioxidant capacity, muffins supplemented with finer particles had higher values. Elasticity and chewability were unchanged, but particle size significantly affected hardness; specifically, hardness increased in samples supplemented with grape pomace of smaller particle size. Sensory analysis revealed that smaller particle sizes negatively affected the samples’ hardness, color, and flavor (astringent and toasted). These changes may have been related to tannins in the grape pomace and lower fiber content. The highest acceptability was obtained on samples supplemented with a pomace of larger particle size (425–300), since it had a better impact on their flavor and smell and greater homogeneity of its structure. Although the present study considered the effects of particle size on various properties of the supplemented food, no potential health effects were analyzed, suggesting the need for further experimentation.

In another study, Baldan, Riveros, Fabani, and Rodriguez [55] used 15 and 25% grape pomace flour to supplement rice flour muffins. They used two drying temperatures on the pomace (55 and 75 °C) and determined that 75 °C was ideal, according to favorable results regarding its physicochemical and nutritional characteristics. The grape pomace supplementation improved the muffins’ characteristics in general; for example, the sample with 25% pomace had a higher moisture content, which the authors propose could be due to its high sugar concentration acting as a humectant. The samples’ lipids, protein, and fiber content increased significantly as the percentage of grape pomace increased. Their sensory analysis revealed an increase in flavor and intensity as the percentage of grape pomace increased; specifically, the panelists preferred the samples supplemented with 15%. Although this study does not report phenolic compounds or antioxidant capacity, they have been generally shown to increase by other authors performing similar experiments [44]. Similarly, no health effects were considered, but other in vivo experiments report favorable health effects when administering a grape pomace extract to hypertensive rats, according to its high content of phenolics in general and anthocyanins in particular [102]. These compounds can also exert antimicrobial effects against some species (*Escherichia coli*, *Bacillus megaterium,* and *Listeria monocytogenes*) [103], potentially exerting other health effects on the consumers, but further experiments are required to demonstrate them conclusively.

Additional studies are required when developing a byproduct-supplemented food since these may contain compounds that can positively affect gastrointestinal or cardiovascular health or other important parameters, but they are commonly not studied in detail. It would also be helpful and interesting to identify and quantify the specific bioactive compounds present on these products as a consequence of being supplemented with fruit byproducts.

## 5. Conclusions

The present work summarized studies where fruit byproducts were used to supplement baked products. It was noted that their addition induces physicochemical changes and can alter their overall acceptability. These changes can be positive if the amount used is carefully optimized with the aid of instrumental analyses and consumer panels but can be negative if this is neglected. Most byproducts have strong flavors and colors, so some authors have considered pretreating them before incorporating them into baked products. The amount added varies according to the specific byproduct used and the specific food (snacks, chips, cakes, or others); it will typically be under 20% since the changes exerted by higher values may overwhelm the final product. Their addition can also induce positive results in terms of their nutritional properties, where an increase in fiber, phenolic compounds, antioxidant capacity, and decreased glycemic index are commonly reported. Some studies perform sensory evaluation and physicochemical analyses that determine the effects of supplementing a given food, which is critical to developing a product with consistent properties. These experiments are sometimes omitted, making it difficult to precisely state the changes imparted to the products and the consumer perception. Analyses to determine the potential effects of the addition of byproducts on consumer health are also absent from most studies. This important parameter should be carefully considered in future research to validate their benefits in vivo. Data on the use of fruit byproducts as ingredients are promising and allow to propose additional studies that consider other kinds of byproducts, particularly those generated locally, to strengthen efforts to minimize food waste and reincorporate it into the processing chain (circular bioeconomy).

## Figures and Tables

**Table 1:** Summary of baked food products supplemented with agro-industrial byproducts and main findings related to the supplementation.

Food Product	Byproduct	Percent Added (%)	Type of Study	Effects	Reference
Biscuits	apple pomace	10 and 20	in vitro and sensory analyses	↑ fiber and phenolic compounds ↓ dialyzed glucose sensory accepted	[41]
	pomegranate peel	7.5	in vitro	↑ fiber, phenolic compounds, and bioaccessibility ↓ dialyzed glucose	[42]
	coffee pulp	10	physicochemical and sensory analyses	↑ fiber, phenolic compounds, and antioxidant capacity sensory accepted	[43]
	grape pomace	6 and 8	physicochemical and sensory analyses	↑ anthocyanins, phenolic compounds, and antioxidant capacity sensory accepted	[44]
	prickly pear peel	20 and 30	physicochemical and sensory analyses	↑ phenolic compounds, fiber, betalains, and carotenoids	[45]
	apple byproducts	15	physicochemical and sensory analyses	↑ fiber	[46]
	pineapple byproducts			↑ fiber sensory accepted	
	melon byproducts			↑ fiber	
	blackberry pomace	3.75	in vitro and sensory analyses	↑ fiber, phenolic compounds, antioxidant capacity, and bioaccessibility sensory accepted	[47]
Corn chips	mango peel	10 and 15	in vitro and sensory analyses	↑ phenolic compounds and bioaccessibility ↓ dialyzed glucose sensory accepted	[48]
	mango peel and roselle byproducts	5 and 10	in vitro and sensory analyses	↑ phenolic compounds and antioxidant capacity ↓ dialyzed glucose sensory accepted	[49]
Extruded snacks and breadsticks	blackcurrant juice press residue	27–28	physicochemical and sensory analyses	↑ expansion ↓ hardness ↑ content of fructose, glucose, and fruit acids sensory accepted	[50]
	mango peel	15	in vitro and sensory analyses	↑ content of phenolic compounds and antioxidant capacity ↑ bioaccesibility sensory accepted	[51]
	papaya peel	15	in vitro and sensory analyses	↑carotenoids ↑ release of bioactive compounds and antioxidant capacity in the intestinal stage	
	red grape pomace	5 and 10	physicochemical and sensory analyses	↑ fiber sensory acceptability ↑phenolic compounds and antioxidant capacity	[52]
Cakes and muffins	apple, orange, and carrot pomace	5, 10 and 15	physicochemical and sensory analyses	↑ fiber ↑viscosity ↑ sensory acceptability of cake supplemented with orange pomace	[53]
	grape pomace	15	Physicochemical and sensory analyses	↑ fiber, protein, and carbohydrates sensory acceptability ↑phenolic compounds and antioxidant capacity ↑total anthocyanins	[54]
	grape pomace	15 and 25	physicochemical and sensory analyses	↑ fiber, protein, and lipids sensory acceptability	[55]

↑ and ↓ indicate an increase or decrease respectively.

## Data Availability

Data sharing is not applicable to this article.
